# Advances in Endovascular Intervention Using Biomaterials: Study on Heat Efficiency of Scissor-Type Ultrasonic Catheter Device

**DOI:** 10.1155/2021/5543520

**Published:** 2021-03-09

**Authors:** Minoru Morita, Jingjing Yang, Zhongwei Jiang

**Affiliations:** ^1^Yamaguchi University, Tokiwadai 2-16-1, Ube, Yamaguchi, Japan; ^2^Kunming University of Science and Technology, 727 South Jingming Road, Chenggong District, Kunming 650500, China

## Abstract

To improve the performance of the ultrasonic device during the endovascular operation, a scissor-type ultrasonic catheter device with compound vibration was developed. The heat generated by friction between the target and the device affects its coagulation mechanism while the actuator contacts the tissue. The scissor-type ultrasonic catheter device proposed in this study is expected to improve heat generation performance because it has the action of rubbing the object when it is pushed by combined vibration. In addition, since it is constructed by simple notch processing, it can be miniaturized and can be expected to be introduced into catheters. However, the observation of ultrasonic vibration during frictional heating is difficult, which is an issue for device design. In this paper, a thermal-structure coupling analysis was done using the finite element method to calculate the heat generation efficiency and evaluate its coagulation performance.

## 1. Introduction

Vibration has been widely utilized for therapeutic purposes. Since the 1950s, many medical devices based on vibration have been invented and applied for therapy applications including tissue cutting, cataract phacoemulsification, fat emulsion, ultrasonography, bone fracture healing, cancer treatment, sonothrombolysis, and so on [1, 2]. These devices work based on mechanical vibration, and the effective vibration needs to be delivered to the tip directly in complicated environments with many restrictions. In this thesis, a new end-effector with the desired vibration mode at the end-effector tip was designed for catheter surgery applications. The proposed new structure of the end-effector can transmit the longitudinal elastic wave through a shaft and convert it into scissor-type vibration (compound longitudinal-transversal vibration) just at the end-effector tip.

The main research line of this thesis is to design an effective end-effector used in a microcatheter for hemostasis by coagulated proteins. In the coagulation of proteins by friction of an end-effector which is excited by mechanical vibration, the tip transversal vibration is needed for supplying friction heating function in narrow blood vessels. An ultrasonic catheter surgery device is a device that uses ultrasonically vibrating heat to denature the tissue protein and simultaneously performs hemostasis and cutting function at the incision. According to the reports on hemostasis by an ultrasonic device [3, 4], the essential hemostatic mechanism is that the coagulated proteins caused by the friction heat seal the bleeding vessels. For these devices, the heat generation efficiency depends on the state of contact between the tissue and the vibrating blade because the blood coagulation needs enough heat (the coagulated protein occurs at 63 degrees [5]). On the contrary, it will take a long time to interrupt the blood flow if the heat does not rise enough, which will lead to incision closure difficulties and other tissue damage. Therefore, we realized that the effective solution for stopping the bleeding quickly during surgery is to improve the heat generation efficiency at the incision.

In this study, we analysed an ultrasonic catheter surgery device with a novel structure to improve the heat generation efficiency by exciting the scissor-type compound vibration at the tip of the device and using the ultrasonic vibration of rubbing while pushing the blade against the tissue. To find the optimal design of this new ultrasonic catheter surgery device, it is necessary to evaluate the heat generation phenomenon and compare it with other models. It is difficult to observe small vibrations at a high sampling speed. Due to the individual differences of biomaterials, the reproducibility of the test and associated parameters which contribute to device performance are difficult to be ensured and evaluated, respectively. Therefore, we developed a finite element analysis model with heat-structural interaction to evaluate the device's coagulating characteristics, and the heat generation performances with different model shapes were simulated and compared in this paper.

## 2. Design and Modelling

### 2.1. Designed Method of Ultrasonic Device

**Figure 1:**
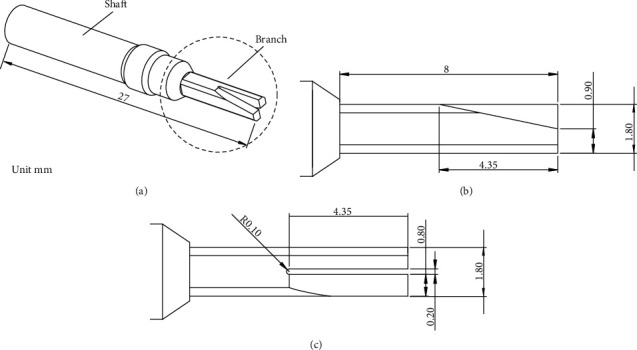
Schematic image of scissor-type ultrasonic device: (a) isometric view, (b) side view of branch, and (c) top view of branch.

The schematic image of the new proposed scissor-type actuator was designed in our previous research [6–9], as shown in Figure 1. This actuator consisted of a shaft and two small branches.

**Figure 2:**
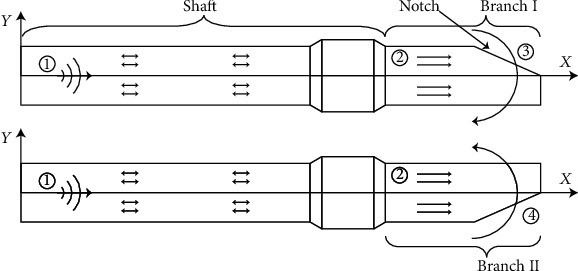
Cutaway of two inverse symmetry branches: ① input signal, ② longitudinal wave propagating to tip, ③ mode conversion and swing branch I, and ④ branch II swings the opposite direction from branch I.

The two branches were connected to the shaft, and two inverse symmetrical slant planes are cut at the head end of the branches.  Figure 2 presents the cutaway view of the scissor-type ultrasonic incision device. It works the following way. Firstly, a sinusoid voltage with the required frequency is applied to the transducer to generate the longitudinal elastic wave. Then, the longitudinal wave propagates through the shaft and reaches to the branch; the wave impinges on the slant plane with an oblique angle and excites a fluctuating motion of the branch due to the reflection and mode conversion caused by the wave reflection. Finally, swing the branches in the opposite direction between branches I and II like a scissor, as shown in Figure 2.

### 2.2. Mathematic Modelling of Structural-Heat Problem

The structural dynamics equation is as follows [10, 11].

(1) $$\begin{array}{c} \left[M\right]\left\{\ddot{u}\right\}+\left[D\right]\left\{\dot{u}\right\}+\left[K\right]\left\{u\right\}=\left\{F\left(t\right)\right\}+\left[P\right]. \end{array}$$

Here, [*M*], [*D*], and [*K*] are the mass matrix, damping matrix, and stiffness matrix, respectively; {*F*(*t*)} is the time-varying load; [*P*] is the contact pressure; and {*u*}, $\left\{\dot{u}\right\},$ and $\left\{\ddot{u}\right\}$ are the displacement, velocity, and acceleration, respectively. The penalty method was used to calculate the contact pressure [*P*], and then Equation (1) was used to calculate the frictional stress and vibrational velocity of the actuator. The transient analysis was done to analyse the heat transfer between the actuator and tissue, as follows:

(2) $$\begin{array}{c} \begin{array}{c} \left[C\right]\left\{\dot{h}\right\}+\left[K\right]\left\{h\right\}=\left\{Q\left(h\right)\right\}, \end{array} \end{array}$$

where [*C*] is the specific heat matrix, [*K*] is the thermal conductance matrix, {*Q*(*t*)} is the heat flow matrix, and {*h*} and $\dot{\left\{h\right\}}$ are the temperature and time derivative of temperature, respectively.

### 2.3. Finite Element Method and Modelling

**Figure 3:**
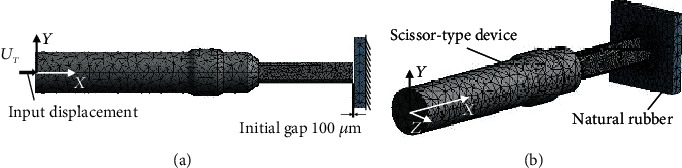
Three-dimensional view of FEM model for scissor-type device. Finite element mesh was generated using 10-node tetrahedral elements: (a) side view; (b) isometric view.

**Figure 4:**
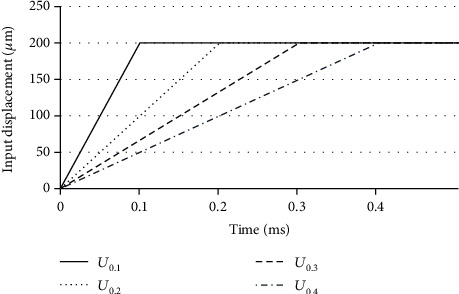
Input displacement signal.

The finite element method (FEM) model used in this study was based on the previous research method, which has proposed an analysis method for friction stir welding [12–18]. The FEM model of the ultrasonic catheter surgery for structural-heat analysis was developed by ANSYS software, as shown in Figure 3. The material of the human tissue was used as the natural rubber to simplify simulation. As the mesh near the contact surface, 0.2 mm 10-node tetrahedral elements were used. The material parameters of the actuator and nature rubber are listed in Table 1. As the boundary condition, the bottom surface of the rectangular parallelepiped rubber target was fixed. From previous research, the shaft of the ultrasonic catheter used in this research has a structure to generate only longitudinal vibration [6, 9]. Therefore, only the *X*-direction displacement was inputted to the left end of the shaft as a boundary condition. The input signal was the displacement with 200 *μ*m added on the left end of the shaft to press the shaft to the rubber target, and the phenomenon of heat generation during vibration of the actuator was recorded. As we know, resonance of the actuator by the input sinusoidal wave requires a relatively long analysis time to obtain enough amplitude, which leads to the problem of computational cost. In addition, the ultrasonic catheter device does not generate heat unless pressed against the target. Considering the above problems, in this study, the input displacement (*U*_*T*_) was sliced by different time, as Equation (3) and Figure 4 show, and enough sinusoidal amplitude and can be obtained in a short analysis time. The initial gap between the catheter device tip and natural rubber was 100 *μ*m.

(3) $$\begin{array}{c} \begin{array}{c} U_{T}=\left\{\begin{array}{c} 200\left[\mu \text{m}\right]∙\frac{\;t}{T}\;\left(t\leq T\right),\\ 200\left[\mu \text{m}\right]\left(t>T\right), \end{array}\right.\;\;T=0.1\sim 0.4. \end{array} \end{array}$$

## 3. Results and Discussion

### 3.1. Effect of Branch Shape

**Figure 5:**
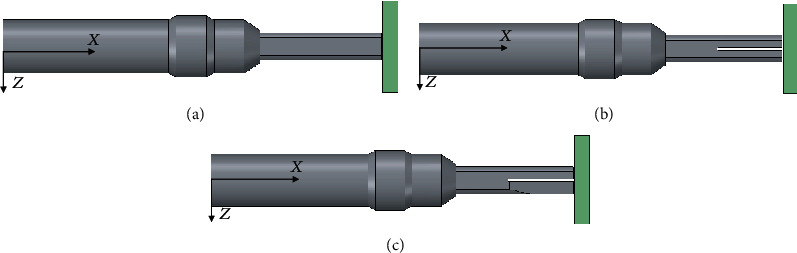
Model shapes of branches with different tips: (a) Model 1 (longitudinal), (b) Model 2 (longitudinal+slit), and (c) Model 3 (scissor).

**Figure 6:**
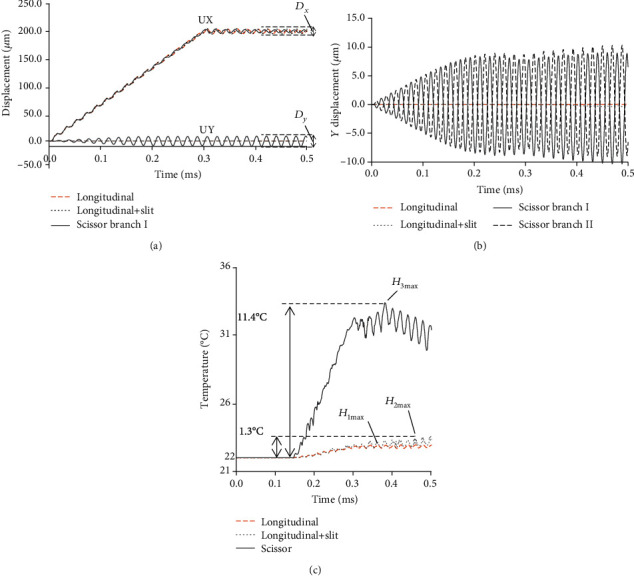
Time curve results of displacement and temperature about each branch model shape tip: (a) *X*- and *Y*-direction displacement-time curve, (b) *Y*-direction displacement-time curve, and (c) temperature-time curve.

To confirm the effect of the branch shape on the heat generation efficiency, three types of models with different structures were developed and analysed, as shown in Figure 5. Model 1 (longitudinal tip) has a simple branch with no slit and notches on the actuator tip, in which the longitudinal vibration mode was excited along the whole actuator. In Model 2 (longitudinal+slit), a slit was cut at the centre of the branch, and the longitudinal vibration mode same as Model 1 was excited. Compared with the above 2 models, Model 3 cut two inverse symmetrical slant planes at the branch tips, and a scissor-type vibration was excited successfully by the longitudinal input signal.  Figure 6 shows the analysis results of the displacement in the *X*- and *Y*-directions and the friction heat temperature for each model. For the input displacement, the function at *T* = 0.3 ms in Equation (3) was used. As the results show, the displacements in the *X*-direction were the same for each model; however, the displacements in the *Y*-direction were totally different. Under the same input longitudinal displacement, the longitudinal vibrations at the branch tips are almost the same, but the transverse vibration of Model 3 was clearly bigger than that of the other two models, which means Model 3 excited the transverse vibration successfully. The *Y*-direction displacements of branches I and II of Model 3 are recorded and shown in Figure 6(b). Obviously, the transverse vibrations of these two branches are reversed at the same time. It is confirmed that each branch vibrated in the opposite direction, which induces a scissor-type vibration at the actuator tip. The displacement in the *Z*-direction was about 10% of the *X*-direction; it is considered that the influence of the heat generation by the *Z*-direction displacement is small.

**Figure 7:**
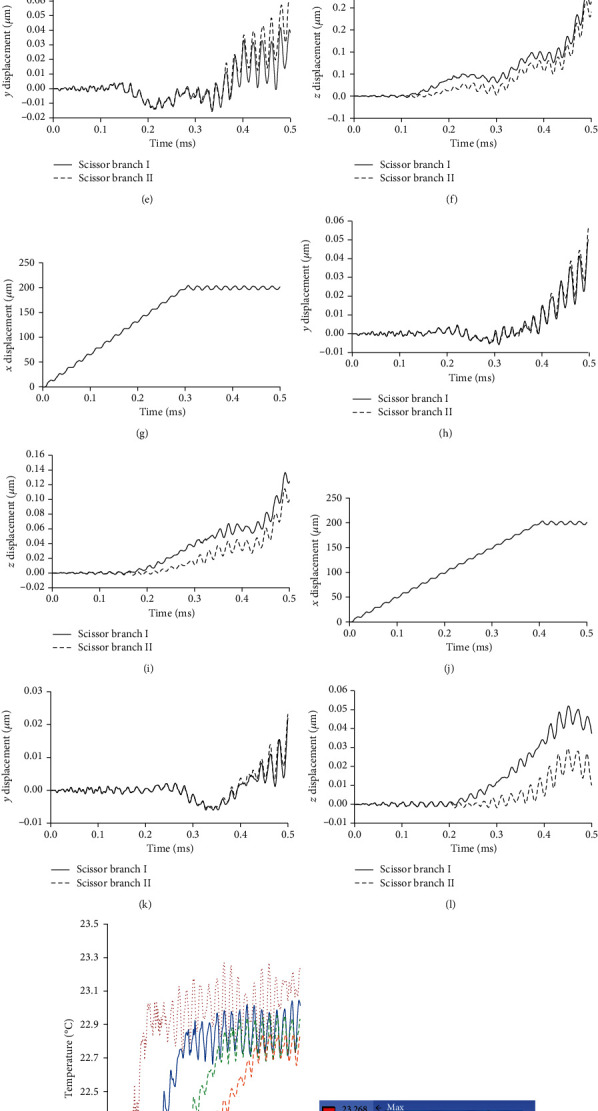
Analysis results of ultrasonic catheter device Model 1 (longitudinal). (a–c) Displacement-time curve of input condition *U*_0.1_. (d–f) Displacement-time curve of input condition *U*_0.2_. (g–i) Displacement-time curve of input condition *U*_0.3_. (j–l) Displacement-time curve of input condition *U*_0.4_. (m) Temperature-time curve. (n–q) Temperature contour map at peak value of input condition *U*_0.1_~*U*_0.4_.

**Figure 8:**
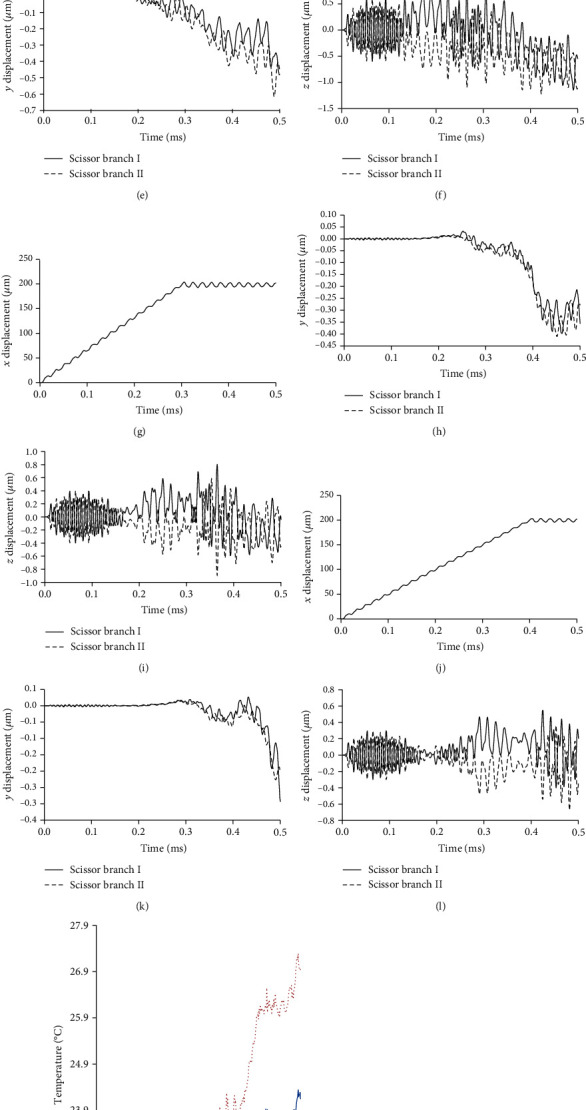
Analysis results of ultrasonic catheter device Model 2 (longitudinal+slit). (a–c) Displacement-time curve of input condition *U*_0.1_. (d–f) Displacement-time curve of input condition *U*_0.2_. (g–i) Displacement-time curve of input condition *U*_0.3_. (j–l) Displacement-time curve of input condition *U*_0.4_. (m) Temperature-time curve. (n–q) Temperature contour map at peak value of input condition *U*_0.1_~*U*_0.4_.

**Figure 9:**
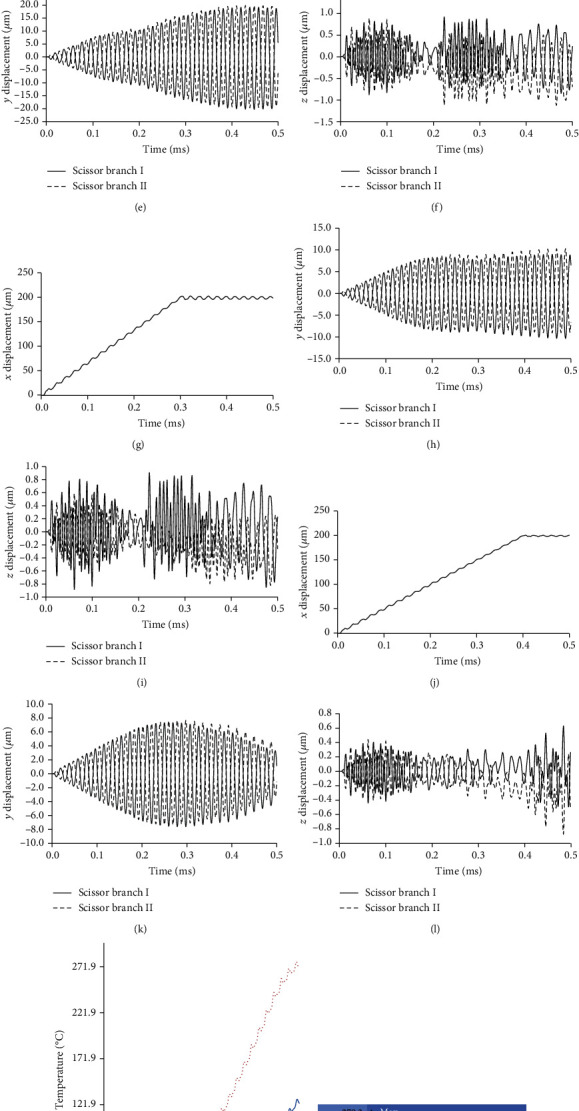
Analysis results of ultrasonic catheter device Model 3 (scissor). (a–c) Displacement-time curve of input condition *U*_0.1_. (d–f) Displacement-time curve of input condition *U*_0.2_. (g–i) Displacement-time curve of input condition *U*_0.3_. (j–l) Displacement-time curve of input condition *U*_0.4_. (m) Temperature-time curve. (n–q) Temperature contour map at peak value of input condition *U*_0.1_~*U*_0.4_.

Comparing the maximum frictional heat temperature (*H*_max_) of each model, Models 1 and 2 increased only 1.3°C from the initial temperature of 22°C in 0.5 ms, while Model 3 increased 11.4°C. The temperature increase rate of the scissor-type actuator is more than 10 times compared with that of the other two models. This indicates that the heating generation of Model 3 is higher than that of the other models. From these results, it is considered that generating the vibration amplitude in the *Y*-direction due to the swing in Figure 2, ③ and ④, contributed to the improvement of the heat generation performance of Model 3. To analyse the thermal distribution between the actuator tip and rubber, the temperature contour maps of different models at the peak value time were recorded and shown in Figures 7–9. The heat generation tends to increase at the edge of the tip of the ultrasonic catheter device.

### 3.2. Effect of Input Velocity

**Figure 10:**
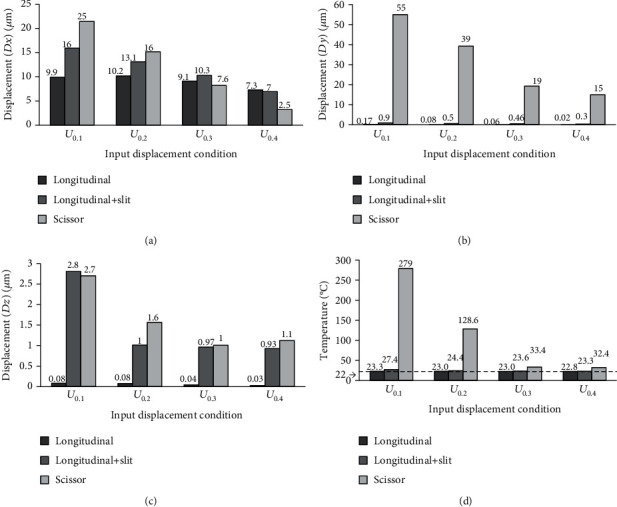
Peak-to-peak displacement and maximum template results of each branch model tip with different input velocities, (a) *X*-direction displacement (*Dx*), (b) *Y*-direction displacement (*Dy*), (c) *Z*-direction displacement (*Dz*), and (d) maximum temperature (*H*max).

Since this paper analyses by the input condition of Equation (3) and Figure 4, the input speed affects the results and the results may differ from the real operation. To evaluate the validity of the analysis model, the effect of changing the input velocity on displacement was discussed in this chapter. The velocity condition was changed by using the function at *T* = 0.1, 0.2, 0.3, and 0.4 ms in Equation (3). The measurement point was set as the catheter device tip. Figures 7–9 show the analysis results about the displacement-time curve, temperature-time curve, and temperature contour map of each model. Figures 10(a)–10(c) show the simulated results of peak-to-peak displacements in each direction (*Dx*, *Dy*, and *Dz*). The results show that the expected vibration modes are excited in each model.  Figure 10(d) summarizes the analysis results of the maximum temperature of the target rubber. The displacement results were suggested to be valid because the displacement in each direction increased with the speed of the input displacement, and the *Y*-direction displacement of Model 3 has the opposite direction vibration in each branch. The temperature results were suggested to be valid because the heat generation starting time has become faster depending on the speed of the input displacement, and the temperature also increased with the speed. In Figure 10(a), the input velocity has a big influence on the output displacement of the *X*-direction in Model 3 (scissor). It is considered that the bending vibration of the *Y*- and *Z*-directions can be affected by the amplitude of the *X*-direction. The dashed line of Figure 10(d) is drawn at the initial temperature of 22°C; the result of the temperature corresponds well to the result of the displacement in the *Y*-direction in Figure 10(b).

Moreover, the result of Model 3 (scissor) suggested that the *Y*-direction displacement has a large effect on the heat generation because the results are better under the input conditions of *U*_0.3_ and *U*_0.4_, even though the *X*-direction displacement is smaller than the other models. From these results, the amplitude increases in a direction parallel (*Y*- or *Z*-direction) to the surface of the target are conjectured to improve the heat generated performance. These results were explained in terms of the heating phenomenon by friction; the validity of the analytical model is considered to have been evaluated. This fact indicates that the scissor-type vibration mode contributes to the improvement of the heat generation performance because this mode vibrates parallel to the surface while pressing the target.

## 4. Conclusions

In this paper, the ultrasound catheter device was designed to generate a composite vibration like a scissor, and then the structural-heat interaction analysis was conducted by a series of finite element models to evaluate the heat generating performance. In the simulation, the results of improving the heat generation performance with our developed scissor-type ultrasonic catheter device were obtained. This fact indicates that the scissor-type vibration mode contributes to the improvement of the heat generation performance because this mode vibrates parallel to the surface while pressing the target. This indicates that the heating generation of our developed ultrasonic catheter device has higher friction heat performance than the other models. We plan in the near future to study the coagulation experiment of blood vessels using a scissor-type ultrasound catheter device.

## Figures and Tables

**Table 1:** Parameters of actuator and natural rubber.

Material property	Titanium	Natural rubber
Young's Modulus	96 GPa	17.6 MPa
Density	4620 kg/m^2^	1200 kg/m^2^
Poisson ratio	0.36	0.3
Thermal expansion	9.4 × 10^−6^/°C	270 × 10^−6^/°C
Thermal conductivity	21.9 W/m°C	0.2 W/m°C
Specific heat	522J/kg°C	150 J/kg°C
Frictional coefficient	0.2	
Initial temperature	22°C	

## Data Availability

The data that support the findings of this study are available from the corresponding author upon reasonable request.

## References

[1] Harr G. T. (1999). Therapeutic ultrasound. *European Journal of Ultrasound*.

[2] Harr G. T. (2007). Therapeutic applications of ultrasound. *Progress in Biophysics & Molecular biology*.

[3] Amaral J. F. (1994). Ultrasonic dissection. *End. Surgery*.

[4] Amaral J. F. (1994). The experimental development of an ultrasonically activated scalpel for laparoscopic use. *Surgical Laparoscopy & Endoscopy*.

[5] Boddy S.-A. M., Ramsay J. W. A., Carter S. S. C., Webster P. J. R., Levison D. A., Whitfield H. N. (1987). Tissue effects of an ultrasonic scalpel for clinical surgical use. *Urological Research*.

[6] Ajoudanian M., Jiang Z. W., Morita M. (2011). Study on a new type micro-stirrer excited by longitudinal elastic wave for thrombus dissolution. *International Journal of Applied Electromagnetics and Mechanics*.

[7] Ajoudanian M., Jiang Z. W., Morita M. (2011). Design of a novel type micro-stirrer excited by longitudinal elastic wave for thrombus dissolution. *Journal of Biomechanical Science and Engineering*.

[8] Ajoudanian M., Jiang Z. W., Morita M. (2013). Structural analysis and design of micro-stirrer driven at a requested frequency for thrombus dissolution. *International Journal of Applied Electromagnetics and Mechanics*.

[9] Yang J., Morita M., Jiang Z. (2016). Design of a novel scissoring micro-stirrer for blood clot dissolution. *Sensors and Actuators A: Physical*.

[10] (2020). *ANSYS workbench mechanical Dynamic analysis seminar text 19.0 (in Japanese) CYBERNET SYSTEMS CO.*.

[11] (2020). *ANSYS workbench mechanical Heat transfer Analysis Seminar Text 19.0 (in Japanese), CYBERNET SYSTEMS CO.*.

[12] Chao Y. J., Qi X., Tang W. (2003). Heat transfer in friction stir welding—experimental and numerical studies. *Journal of manufacturing science and engineering*.

[13] Zhu X. K., Chao Y. J. (2004). Numerical simulation of transient temperature and residual stresses in friction stir welding of 304L stainless steel. *Journal of Materials Processing Technology*.

[14] Soundararajan V., Zekovic S., Kovacevic R. (2005). Thermo-mechanical model with adaptive boundary conditions for friction stir welding of Al 6061. *International Journal of Machine Tools and Manufacture*.

[15] Prasanna P., Rao B. S., Rao G. K. M. (2010). inite element modeling for maximum temperature in friction stir welding and its validation. *The International Journal of Advanced Manufacturing Technology*.

[16] Balcı M. N., Yıldırım B., Dag S. (2015). Analysis of frictional contacts with heat generation considering temperature dependent properties. *International Journal of Mechanical Sciences*.

[17] Zhang C. S., Feng F. Z., Min Q. X., Zhu J. Z. (2015). Effect of engagement force on vibration characteristics and frictional heating in sonic IR imaging. *NDT & E International*.

[18] Chen G., Ma Q., Zhang S., Wu J., Zhang G., Shi Q. (2018). Computational fluid dynamics simulation of friction stir welding: a comparative study on different frictional boundary conditions. *Journal of Materials Science & Technology*.

